# Sensitizing Family Caregivers to Influence Treatment Compliance among Elderly Neglected Patients—A 2-Year Longitudinal Study Outcome in Completely Edentulous Patients

**DOI:** 10.3390/healthcare9050533

**Published:** 2021-05-02

**Authors:** Mohammed A. Alqarni, Khurshid Mattoo, Surbhi Dhingra, Suheel Manzoor Baba, Fuad Al Sanabani, Bandar M. A. Al Makramani, Hadeel Mohammed Akkam

**Affiliations:** 1 Department of Restorative Dental Sciences, College of Dentistry, King Khalid University, Abha 61421, Saudi Arabia; maalqarny@kku.edu.sa (M.A.A.); baba@kku.edu.sa (S.M.B.); 2 Department of Prosthetic Dental Sciences, College of Dentistry, Jazan University, Jazan 45142, Saudi Arabia; fuad_ali2000@yahoo.com (F.A.S.); makramani@yahoo.com (B.M.A.A.M.); Hd761@hotmail.com (H.M.A.); 3 Department of Oral Maxillofacial Prosthetics, Subharti Dental College, Swami Vivekananda Subharti Univeristy, Meerut 250001, India; drsurbhidhingra99@gmail.com

**Keywords:** elder maltreatment, compliance, caregivers neglect, abuse, patient education

## Abstract

Healthcare workers have reported a certain segment of geriatric patients that are suffering from abuse/neglect, which in turn has been associated with anxiety, depression, and helplessness in the individual. Family caregivers (blood relations), being the most common perpetrators of elder abuse and neglect (EAN), have also been shown to respond to sensitization if the type of EAN and the interventions are appropriate. This study was aimed to comparatively analyze the influence of intervention (psychotherapeutic sensitization of FCG) upon long-term (24 months) treatment maintenance and satisfaction in elderly neglected patients. One hundred and fifty patients (aged 41–80 years) suffering from elder neglect (EN) (self-confession) and their respective FCGs, fulfilling the study criteria, participated in this longitudinal 2-year study. The patients were randomly distributed (simple random, convenient) in two equal groups (75 each), namely Group (GP) A (control) and GP B (test). A standardized, complete denture treatment was initiated for all the participants. Both the FCGs and the patients of GP B were sensitized (psychotherapeutic education) for EN, while there was no such intervention in GP A. The influence of such intervention was measured for denture maintenance [denture plaque index (DPI) scores] and treatment satisfaction (10-point visual analog scale). Absolute/relative frequencies and means were major calculations during data analysis. Differences between the groups for any treatment compliance parameter was done through the unpaired *t*-test, while Karl Pearson’s test determined the level of relationship between variables (*p*-value < 0.05). Decrease in mean DPI scores (suggesting improvement) was seen among patients in GP A from 1 month (m = 2.92) to 24 months (m = 2.77). A negligible increase in DPI scores was observed among patients of GP B from 1 month (m = 1.38) to 24 months (m = 1.44). Differences in mean values between the two groups were statistically significant at 24-month intervals, while the relationship between the variables was nonsignificant. FCG sensitization through psychotherapeutic education shows a long-term positive influence on the treatment compliance (maintenance and satisfaction). Identifying the existence of EAN among geriatric patients, followed by psychotherapeutic education of FCGs is recommended for routine medical and dental long-duration treatment procedures.

## 1. Introduction

Humans have always been fascinated by life and death, and across various civilizations, efforts have been made to conquer both. Increased life expectancy and the advent of cloning are two characteristic examples of how life is treasured by humans. Increased life expectancy has not, though, achieved a prolonged, youthful age. On the contrary, it has definitely increased the geriatric population of the world. Projections for developed nations such as the United States and developing countries such as India predict that elderly people will constitute about 20% and 12% of their entire population respectively by the year 2050 [1,2]. Taking care of such vast elderly populations in the future is going to be a challenge, especially in sensitive and critical social issues such as elder abuse and neglect (EAN). Conflict between parents and children is as old as humanity itself, but modern reports of EAN in medical literature date back to 1975, with the then-famous publishing of a paper on “granny battering” in the British Medical Journal [3]. Most developed nations at present recognize its presence and consequences, for many medical organizations (American Medical Association and Council of Europe), have defined the terms to comprehend its legality within the framework of the constitutions and legislations of various governments [4]. Much of the emphasis within these frameworks signifies the role and relationship between the caregiver and the caretaker [5]. A recent estimate (52 studies in 28 countries) of nearly 16% of elderly persons (>60 years) being subjected to some form of abuse marks its presence in various societies globally [6]. EAN has been reported to be as low as 1.1% to as high as 44.6% in various populations, with Asian countries sharing higher percentages than western countries [7]. Elder abuse (EA) exists in multiple forms, such as neglect, emotional/psychological, financial, sexual, and physical. Elder neglect (EN) has been widely reported as the most common and one of the most ubiquitous [7] and most concealed forms of EA [8]. 

EN in any of its forms varies widely among nations, societies, cultures, and even religions. Studies have reported its occurrence in settings such as hospitals (nurses or staff abusing the elderly), elder homes (staff/caretakers abusing the elderly), and, most commonly at in the home within an elderly person’s own family (son, daughter, spouse) [9]. Most cultures across the world expect children to care for their elderly parents when they grow older; however, job and growth opportunities for individuals may hamper their zeal for caring for the elderly. Yet, in some cases, it may be done deliberately as part of intimidation or revenge. Thus, EN in many cases can be circumstantial, but in most cases it is deliberate [8,10]. Elder caregiving has been found to be physically and emotionally demanding for caregivers [11]. Despite the stress involved, the family caregivers are expected to provide unconditional care for the elderly. EN occurring within a family can be difficult to detect, but healthcare workers (physicians, dentists, nurses) are not only trained to do this, but also are seen to develop trust-building relationships with their patients, especially with long-term medical and dental procedures [12]. EN in the prevalence range of 30 to 40% has been reported among patients visiting medical [13] and/or dental [14] hospitals/colleges (outpatient departments). Victims of EN have been reported to have a higher risk of an adverse health outcome (depression, disability), ref. [15] and an increased risk for mortality [16], and these can be potential markers in futuristic forensic science [17]. Depression is the most common psychological condition that has been reported to be present among elders, especially those who are suffering from EN [18]. The presence of depression among patients has been associated with noncompliance with both medical [19] and dental treatment [20]. Therefore, healthcare workers (in particular doctors) should consider EN to have widely different, dual applications in their daily practice. It must be, however, reiterated, that studies in the field are extremely scarce in medical and dental fields, despite the topic being general to all specialties and subspecialties. Besides detecting such underlying influences in their patients, they must also identify the short- and the long-term influences of this condition on their respective medical/dental treatment compliance. Wherever appropriate, interventions in terms of supportive, service-based psychoeducation and psychotherapy of caregivers are recommended [14]. While the role of psychotherapeutic education of family caregivers has demonstrated a positive influence upon short-term complete denture treatment compliance, ref. [14] this longitudinal comparative case control study is a further continuation of the same study and it intends to analyze the long-term influence of one-time psychotherapeutic education sessions of family caregivers. Depending upon the outcome, one may then decide the need for further sensitization of family caregivers in long-term medical and dental treatments. We hypothesized that a single psychotherapeutic education session for a family caregiver will continue to influence elderly patients to develop positive treatment compliance. Differences in denture maintenance and treatment satisfaction will continue to exist among patients who received the intervention (dependent variable) with those who did not receive any intervention (independent variable). 

## 2. Materials and Methods

Ethics: The essential data collection of this study was performed on routine geriatric patients visiting an outpatient department (OPD) of the geriatric care unit of the department of oral maxillofacial prosthetics in one of the longest-established and most-recognized universities of Northern India. All studies done on humans and animals are required to maintain the ethical standards as laid down in the Helsinki declaration after getting the approval of the university ethics committee. Subjects who participated in the study were informed about the beneficence, nonmaleficence, right to withdraw, manner of conductance, confidentiality, and anonymity of the study before they gave their informed consent. 

Study design: This comparative, case-control longitudinal study based on an informal experimental design was conducted between September 2017 and January 2020, on a randomly selected geriatric population (North India, aged 41 to 80 years). The study is essentially the second phase (part) of an earlier published study, which reported the short-term impact of family caregiver sensitization among EN patients upon treatment compliance (denture satisfaction and maintenance) [14]. Since the methodology essentially remains the same, a brief summary will be provided to build the context for the present study. The whole study was conducted in three different stages (stage 1—large-scale survey to derive the sample (convenient), stage 2—complete denture treatment and psychotherapeutic education of FCGs, stage 3—short-term (one month) and long-term (2 years) follow-up).

Operational definition: EN was defined as the failure of the family caregiver to meet the basic needs of an elderly individual, which could range from providing food, water, shelter, hygiene, and essential/nonessential medical care [21]. Since EN is a type of elder abuse, the terms in the article are used interchangeably and inclusively, since most of the research does not differentiate the two as separate entities. A family caregiver is defined as the one, who as a result of family relationship (blood related) is accountable for caring for the concerned elderly patient. Caregiver neglect is defined as a specific mistreatment in which the FCG intentionally fails to address the physical, social, or emotional needs of the elderly. 

Sample preparation, selection, and grouping: This study was conducted on two types of participants, namely completely edentulous patients and their respective family caregivers. All completely edentulous patients were screened by a four-member team (psychiatrist, clinical psychologist, physician, prosthodontist) for cognitive impairment [Elderly Cognitive Assessment Questionnaire (ECAQ)], ref. [22] followed by screening for EAN [Elder Abuse Suspicion Index (EASI)] [23]. Those who were suspected of suffering from EN were further screened to fulfill the criteria particular to the study. Patients that were seeking CD treatment for the first time; were living with family (children); were potentially cooperative; shared personal sensitive information willingly; and did not have systemic diseases, sensory impairment, or drug/alcohol abuse problems was considered eligible for the study. Inclusion criteria for the FCG were that he/she should accompany the patient at all/most times, have no history of behavioral disorder or drug/alcohol abuse, should not be burdened with the caring demands of other immediate family (chronic sickness of spouse/child/in-laws), be financially independent, and did not have a history of being neglected by his or her parents when he or she was a child. All participants who showed signs of self-neglect, who were childless or had lost a child at some time, had poor, completely edentulous foundations, poor neuro-muscular control and coordination, and those who would withdraw from long-term study without prior notice were completely excluded from the study. All clinicians who would be treating the patients in the study were first trained in measures to build trust with their respective patients to elicit details of existing elder abuse of any form. Clinical/non clinical/laboratory procedures for fabricating the complete denture prosthesis were standardized for appointments, denture designs, and follow up protocol. A total of 150 confirmed EN patients (aged 45 to 80 years) were divided into 2 groups, namely Group (GP) A (Control GP (FCG not sensitized)) and GP B (Test GP (FCG sensitized)). To minimize the influence of gender, equal number of males and females were randomly distributed in each group (64 males, 11 females). Sample design for both groups was thus convenient (consecutive) sampling (convenience being EN) while distribution was random. Treatment procedures for each patient was done under the same doctor and supervisor, while the evaluation was double-blinded in terms of evaluators and/or doctors. FCG sensitization through psychotherapeutic education was done for patients and their respective FCGs in GP B. Counselling of both was based on the principles as laid down in the earlier study [14]. FCG counselling identified areas of caregiving stress, and methods to cope with them was described to individual FCGs. The first counselling session gathered maximum information about their relationship, while the last session briefed about the limitations of an elderly patient in terms of effective treatment compliance and the role of the FCG to help the patient overcome those limitations. Principally the aim was to eliminate the neglect that the patient was suffering from, which would indirectly influence the treatment compliance in return. The FCG was empowered with the responsibility in helping the doctor to change the patients’ lifestyles and mental attitudes towards prosthetic care. Utmost precautions were taken not to harm the relationship between the elderly person and his or her FCG. Written instructions (electronically typed) regarding the use and maintenance of dentures were given to the patient after verbally explaining each instruction individually. All patients in both groups also were provided with a denture maintenance kit (brush, denture cleanser, box) to enhance treatment compliance. At the end of 1 month and again after 2 years, the patients in both groups were recalled for assessing denture satisfaction and denture maintenance. 

Measures, data evaluation, collection, and analysis: A valid and reliable, self-administered, structured questionnaire was used to collect the demographic characteristics. Different linguistic versions were prepared for the subjects’ clear understanding. A record diary was maintained by each treating doctor in which they noted information relevant to EN, with which their patients would provide them during the course of appointments. The denture plaque index (DPI) was used to evaluate denture hygiene (which also indirectly measures patients’ motivation and compliance) scores [24]. Treatment satisfaction was evaluated using a questionnaire that quantified the level of treatment satisfaction on a 10-point visual analog scale (VAS) [25]. Data regarding both parameters was collected at 1-month and 24-month intervals. Collected data were studied, reviewed, refined, coded (supplementary data), and then entered on the Statistical Package of Social Sciences (SPSS 25.0) (IBM, Armonk, NY, U.S.) software. Frequency distribution, and mean values with standard deviations defined continuous and qualitative variables. Parametric testing (i.e., the independent *t*-test) was used to examine differences in responses between the two groups where data were normal. Non-parametric testing was used for non-normal and ordinal data. The unpaired *t* test was used to determine the difference between two groups for denture maintenance and various parameters of denture satisfaction. The Karl Pearson correlation coefficient determined the level of relationships between related variables (linear relation) at both intervals of time. All differences were considered to be statistically significant when the probability value (*p* value) would be less than 0.05. 

## 3. Results

The present study involved a long-term (24 months) evaluation of the influence of each FCG’s sensitization through psychotherapeutic education. The collected data at 24 months was also compared with the data collected at the 1-month interval. The comparative demographics of patients and their FCGs in both groups, in terms of various characteristics and parameters are presented in Table 1. Average age of the elderly patients was 63.62 (GP A (64.9), GP B (62.5)) years while that of the FCGs was 28.86 (GP A (30.05), GP B (27.86)). Most of the FCGs belonged to the age range of 21 to 40 years. All subjects in both groups were confirmed cases of EN (self-acceptance). Around 26.6% (GP A (21.3%), GP B (32%)), were observed to suffer from other types of EA, with neglect being the chief type of EA. A higher frequency of illiterate patients was in GP B (90.6%) than GP A (82.6%), while the differences between the level of literacy was negligible among FCGs between both groups. Higher frequency of family caregiving was provided by a son (55.3%) and daughter-in-law (32.6%) with negligible differences in distribution between both groups. In both groups, the higher frequency of FCGs consisted of those who had a low income level (Atlas method-World Bank). Self-rated health shows that higher frequencies of elders (88%) and FCGs (92.6%) were overall healthy. 

Denture maintenance evaluation: The mean scores for the denture plaque index (DPI) and the statistical significance of the differences between the two groups at 1-month and 24-month time intervals are shown in Table 2. While higher frequency of a score of 1 (indicating good denture hygiene) was observed in patients belonging to GP B, there was an almost equal distribution of average, poor, and very poor scores in GP A. Improved denture maintenance was observed in patients of GP A (control) from 1-month scores (2.92) to 24 months (2.77), while there was a negligible decline among patients in GP B from 1 month (1.38) to 24 months (1.44). Differences in means between groups were found to be statistically significant (using the unpaired ‘*t*’ test) at the probable value (*p* < 0.05), at both time intervals (1 month and 24 months). However, there was no correlation found between the two variables (Karl Pearson) suggesting that any change in the values of the independent variable did not cause any proportional linear change in the values of the dependent variable and vice versa. In other words, the two variables did not show any correlation. 

**Figure 1:**
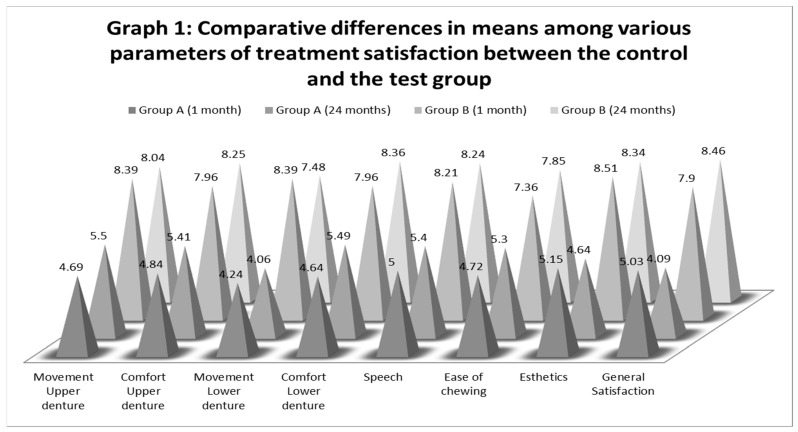
Graphical comparisons among various parameters of treatment satisfaction between the studied groups.

Treatment satisfaction evaluation: Long-term patient satisfaction with the denture treatment was analyzed for eight parameters (movement in both maxillary and mandibular dentures), comfort (in both maxillary and mandibular dentures), speech (phonetics), esthetics, ease of mastication, and general satisfaction (overall). Within GP A, three parameters (movement of mandibular denture, esthetics, and general satisfaction) showed a decline in means at 24 months as shown in Table 3, while the remaining five saw an increase in mean scores. Within GP B, the three parameters of treatment satisfaction, namely movement in both maxillary and mandibular dentures, and esthetics, showed a decrease in mean values, while the remaining five parameters saw an increase between the 1-month and 24-month evaluation (Table 3). The differences between the mean scores of 1 month and 24 months, for both treatment-compliance parameters (maintenance and satisfaction), however, were not statistically significant. Mean scores for all parameters of denture satisfaction, however, were statistically significant for the differences that occurred between GP A and GP B (*p* <0.05). The degree of correlation between linearly related variables at two intervals of time (1 and 24 months) was significant for four parameters (movement of mandibular denture, ease of chewing, esthetics, and general satisfaction. For each parameter of treatment satisfaction, the differences in means at two intervals of time are graphically demonstrated in Figure 1. 

## 4. Discussion

This study was accomplished in two different clinical stages. In the first stage, EN patients were identified and then treated for complete denture prosthetic treatment, during which their respective FCGs were sensitized (psychotherapeutic education) and the short-term (1 month) influence of this variable on treatment compliance was measured for denture maintenance and treatment satisfaction [14]. The present study (second stage) aims to analyze the longitudinal (24 months) influence of FCG sensitization and, based on the obtained results, plan future studies. This study reports that one-time FCG sensitization has a positive influence on treatment compliance even at the end of 2 years. Main findings include continuation of denture hygiene maintenance among patients in GP B (test) after 24 months (m = 1.44). Although the subjects of GP A (control) also showed improved denture hygiene scores (1 month (m = 2.92) and 24 months (m = 2.77)), the differences in the denture hygiene scores at 24 months stood statistically significant (*p* < 0.05). Moreover, their DPI scores also continued to remain in the poor category as per the DPI Index [24]. For treatment satisfaction, three parameters showed a decline in means between 1 month and 24 months (movement of maxillary and mandibular dentures, esthetics) among patients of GP B (test), but still the differences in means were statistically significant between the two groups. Overall, results indicate that neglected elderly patients whose FCGs were sensitized and empowered during the course of treatment, maintain their improved treatment compliance over a period of 2 years. 

Abuse among human beings is ancient and has been socially accepted as a method to instill discipline within a family. A global population rise with increased life expectancy will definitely challenge the caregiving of normal, healthy elders, let alone those who are totally dependent on caregivers because of systemic conditions. The burden of elder care in the near future is primarily going to rest on the families of the elderly, especially in developing or underdeveloped nations. EN is one of the most common and widely reported forms of EA [26]. A study has reported that 16% of persons in general (≥60 years) are suffering from EA [6], and estimates as low as 1.1% and as high as 44% are suffering from EN, with prevalence in the subcontinent higher than in the west [7]. Surprisingly, the prevalence of EA has been observed to be high in the dental outpatient department (40%) [27] and medical emergency (55%) [13]. Such high prevalence in OPDs has prompted professionals to rethink the matter—that more should be done about EN rather than just reporting its existence [14]. Besides being a professional responsibility (identification/intervention) [28], government authorities have made it an accreditation requirement for hospitals [28] and have urged family physicians and family dentists to actively participate in identification and intervention [29]. In severe cases, hospital screening has been found to be the only source where the elderly may have contact with caregivers outside of their residence [30]. It is also significant for healthcare workers to know that both doctors and nurses have been found to be seriously deficient in their knowledge of indicators of EA [31], despite nurses being in greater contact time with elders [32].

### 4.1. Impact on Treatment Compliance

EA in general has been associated with a high risk of developing depression [18], dissatisfaction with life (feeling of helpless and worthless) [16], and when it occurs for a long time, developing other adverse systemic health problems such as anxiety or heart problems (high blood pressure) [33]. Results from this study show that at the end of 2 years of denture wearing, there was only a slight improvement in patients’ DPI (difference of 0.15) score among patients in GP A (no intervention). Improvement in DPI score was still poor at the end of 2 years in GP A. The results agree with studies that have found poor oral hygiene (root caries in dentulous patients) in EN individuals that were unable to achieve minimum oral self-care [34]. An important aspect of denture hygiene maintenance as part of accomplishing long-term treatment compliance is the cognitive and/or functional impairment in an elderly individual. In such cases, poor denture hygiene has been shown to be due to unintentional caregiver neglect, since the caregivers are not aware of the elder’s needs for denture hygiene [35]. This also explains the importance of screening patients for cognitive impairment while selecting the sample, as done in this study. Another important parameter that has been associated with poor oral hygiene is when the elders are uncooperative [36], but such behavior has been studied only in institutionalized patients and not with FCGs at home. Results from this study also reveal that at the end of the 24 months’ evaluation of denture maintenance, the patients in GP B (with intervention), continued to demonstrate a low DPI score, thus rating their maintenance as very good on the DPI scale. It might be recalled that the FCGs of the EN patients in GP B were sensitized through one-time psychotherapeutic education [14]. Our results also agree with studies that have concluded the need for education-based interventions, which have shown improvements in the knowledge, recognition, and prevention of EA [37]. Frequent and substantial emphasis by various authors studying EA have repeatedly urged healthcare professionals to develop, evaluate, and scale potentially effective prevention intervention strategies for EA [38,39]. Educational interventions for caregivers in healthcare settings that include family-based interventions have been reported on elders suffering from various abuses [40]. Most studies, however, are primarily directed at preventing or reducing abuse through population education. 

Most medical and dental treatments are considered successful if the patient is satisfied with the outcome of the treatment. Perhaps denture treatment is one of the rare treatments where the results of the treatment can be gauged by the patient immediately within a day of functioning. Patients’ mental attitude towards treatment, their cooperation, and psychological wellbeing are some of the patient-related factors that influence treatment outcome and treatment satisfaction. Treatment satisfaction with complete denture prosthesis is complex and it must be evaluated only after long-time denture wearing. Treatment satisfaction was measured on eight different parameters for subjects in both groups. For patients in GP A (no intervention), five parameters showed improvement (negligible on a scale though) between 1-month and 24-month evaluations, while three parameters (movement of mandibular denture, esthetics, and general satisfaction) showed a decline in mean values at 24-month intervals. On the other hand, the patients in GP B (intervention) continued to maintain and/or show improvement in their five parameters (comfort maxillary and mandibular denture, speech, ease of chewing, and general satisfaction) of treatment satisfaction. Decline in mean scores (negligible, decimal value) was observed among the three parameters (movement of maxillary denture, movement of mandibular denture and esthetics). The differences in mean scores between the two groups at two time-intervals were statistically significant for all parameters of treatment satisfaction, suggesting that FCG sensitization improves patient treatment compliance. The linear relationship between two variables at different intervals (1 month and 24 months) was positively related for four parameters (movement of mandibular denture, ease of chewing, esthetics and general satisfaction). During the second evaluation, patients in GP A were observed to be nonadherent to the instructions that were given to them at the time of prosthesis delivery. Noncompliance with treatment has been reported to be one of the behavioral mediators found in patients suffering from anxiety and depression [41]. Depression, at the same time, has been found to be strongly associated with denture dissatisfaction [14,20]. With every unit increase on a depression scale, the probability of dissatisfaction has been reported to be around 24% (95% confidence interval, 15–34%, *p* < 0.001 (simple logistic regression)). In another study, depression was a significant risk factor on treatment adherence, with odds of nonadherence being three times higher than in nondepressed patients [19]. EN patients who have underlying health problems are more likely to suffer from depression that is related to the disease [42] under normal physiological conditions; completely edentulous patients may not have treatment satisfaction early during the adaptation period. Not being satisfied after 24 months, despite not seeking new dentures or not complaining about it is routinely linked to a behavioral issue (psychological) rather than technical. Both underlying anxiety and/or depression complicate other treatment, the extent of which and the reasons are still not well-understood [43].

### 4.2. FCGs Sensitization and Long-Term Family Caregiving

The interrelation between the caregiver and the care recipient has been considered to be interdependent but complex at various times [44]. EA has also been considered to occur at highly unpredictable intervals, therefore risk factors (static and dynamic) have been suggested, especially when physical abuse takes place [45]. While static factors are thought to be difficult to change, the dynamic factors have been termed as flexible and can be modified/changed through short- or long-term interventions [46]. Maintaining the influence of FCG sensitization for a long time was ensured by preparing a list of instructions that were based on the various principles and domains of caregiving (Table 4) [47,48,49,50]. While the FCG was educated about the positive effects of caregiving (especially bringing caregiving closer to the elderly patient), the elderly patient was educated about the negative effects that caregiving can have on the FCG [51].

### 4.3. Strength and Limitations of the Study

This study is perhaps the first to explore the possibility of long-term intervention (psychotherapeutic education) of FCGs upon the treatment compliance of neglected, elderly, completely edentulous patients. Since the study has been conducted on a carefully selected sample of EN patients, the inferences may not be applicable to EA in general. Every medical and dental treatment is individualized and unique, and differ in terms of treatment compliance for each patient; therefore, various treatment compliances may be affected differently. The study also highlights that EN does influence the outcome of denture treatment in both short and long terms. The study’s strength lies in that all neglected cases had confirmed and confessed their sufferings at the hand of their caregivers, thus eliminating the possibility of confounding variables. Whether the EN was intentional or nonintentional is a matter of future study. The study is limited in that the study was conducted in FCGs only and not professionals such as nurses, staff, etc., in a hospital. Limitations that are inherent in cross-sectional studies also apply to this study.

## 5. Conclusions

A one-time psychotherapeutic education to sensitize the FCGs of elderly, neglected, complete-denture patients influences the parameters of denture maintenance and treatment satisfaction. The positive influence was observed at 1 month (short-term) and continued to influence till 24 months (long-term) without the necessity of further intervention. Since a single complete denture may last for 5 years, while implant-supported complete dentures may last for 10 years or more, the long-term influence in such cases needs to be studied. Identifying elder neglect among patients should be taught in medical and dental schools at all levels (undergraduate, postgraduate, doctoral).

This study also brings a number of policy, practice, and future guideline issues, which are not clearly mentioned by the concerned central and state governments. As compared to many developed countries, where reporting of EAN to concerned authorities is mandatory, there are no clear or defined laws in developing countries such as India. Current laws and regulations (the Maintenance and Welfare of Parents and Senior Citizens Act 2007) requires the suffering elderly person to report to concerned authorities, which are also not well-defined. Active screening of the elderly in community-based health centers is essential to isolate the victims, which can be used to establish programs for intervention strategies.

## Figures and Tables

**Table 1:** Group comparison of sociodemographic characteristic distribution in the studied groups.

Characteristic	Parameter		Total Subjects *N* = 150	Sample Subjects (N/%)	
				Group A (Control)	Group B (Test)
			*N* (%)	*N* = 75	*N* = 75
Gender distribution	Male		128 (85.33)	64 (85.33)	64 (85.33)
	Female		22 (14.67)	11 (14.67)	11 (14.67)
Age distribution	Elderly	41–50	4 (2.67)	1 (1.33)	3 (4)
		51–60	58 (38.66)	26 (34.66)	32 (42.67)
		61–70	46 (30.66)	26 (34.66)	20 (26.67)
		71–80	42 (28)	22 (29.33)	20 (26.67)
	Family caregiver	≤20	11 (7.34)	5 (6.67)	6 (8)
		21–30	75 (50)	31 (41.33)	44 (58.6)
		31–40	54 (36)	34 (45.33)	20 (26.67)
		41–50	6 (4)	4 (5.3)	2 (2.66)
		≥51	4 (2.66)	1 (1.33)	3 (4)
Type of abuse	Neglect		110 (73.34)	59 (78.66)	51 (68)
	Combination (neglect + one or more)		40 (26.66)	16 (21.33)	24 (32)
Education	Elderly	Illiterate	130 (86.67)	62 (82.67)	68 (90.67)
		Literate	20 (13.33)	13 (17.33)	7 (9.33)
	Family caregiver	Illiterate	116 (77.33)	57 (76)	59 (78.67)
		Literate	34 (22.67)	18 (24)	16 (21.34)
Family caregiver (Types)	Son		83 (55.33)	41 (54.66)	42 (56)
	Daughter in law		49 (32.66)	26 (34.66)	23 (30.6)
	Spouse		14 (9.33)	8 (10.67)	6 (8)
	Sibling		2 (1.33)	0 (0)	2 (2.66)
	Others		2 (1.33)	0(0)	2 (2.66)
Income of family caregiver *	Low		116 (77.34)	56 (74.6)	50 (66.67)
	Average		32 (21.33)	12 (16)	20 (26.67)
	High		12 (8)	7 (9.34)	5 (6.67)
Self-rated health	Elderly	Unhealthy	18 (12)	3 (4)	15 (20)
		Healthy	132 (88)	72 (96)	60 (80)
	Family caregiver	Unhealthy	11 (7.33)	5 (6.66)	6 (8)
		Healthy	139 (92.67)	70 (93.33)	69 (92)

N—Number of subjects; %—Value expressed in terms of percentage within parenthesis; *—Income described as per World Bank country classification (Atlas method).

**Table 2:** Frequency distribution of various grades of DPI (denture plaque index) scores with means compared among subjects of both groups.

		Frequency DistributionN (%)				Mean ± SD				Unpaired “*t*” Test	Karl Pearsons Correlation Coefficient
S.No.	Scores	GP A (Control) (N = 75)		GP B (Test) (N = 75)		GP A (Control) (N = 75)		GP B (Test) (N = 75)			
		1 M	24 M	1 M	24 M	1 M	24 M	1 M	24 M	Probable Value	(r)
1	Good	4 (5.3)	9 (12)	52 (69.33)	50 (66.67)	2.92 ± 0.892	2.77 ± 0.887	1.38 ± 0.618	1.44 ± 1.187	0.0000 *	−0.0012 (NS)
2	Average	23 (30.6)	20 (26.67)	18 (24)	18 (24)						
3	Poor	27 (36)	26 (34.67)	5 (6.67)	7 (9.33)						
4	Very Poor	21 (28)	20 (26.67)	0 (0)	0						

Abbreviations: N = Number, M = number of months, SD = Standard deviation, % = percentage, GP (Group); * denotes statistically significant differences between two groups at the value of (*p* < 0.05); NS = not significant. In the unpaired ‘*t*’ test the level of the degree of significance was determined on the value of *p* ˂ 0. 05. Degree of linear relationship between two variables was determined by the Karl Pearson correlation coefficient expressed as *r*.

**Table 3:** Comparative differences in means among various parameters for denture satisfaction at two different intervals of time between the studied groups.

S.No.	Denture Parameters	GP A (Control)		GP B (Test)		Unpaired “*t*” Test Values	Karl Pearsons Correlation Coefficient
		Mean ± SD (1 M)	Mean ± SD (24 M)	Mean ± SD (1 M)	Mean ± SD (24 M)	Probable Value	R Value
1.	Movement maxillary denture	4.69 ± 1.286	5.50 ± 1.674	8.39 ± 0.658	8.04 ± 0.875	0.0000 *	0.1614
2.	Comfort maxillary denture	4.84 ± 1.372	5.41 ± 0.988	7.96 ± 0.918	8.25 ± 0.978	0.0000 *	0.1451
3.	Movement mandibular denture	4.24 ± 1.031	4.06 ± 1.183	8.39 ± 0.658	7.48 ± 1.122	0.0000 *	0.0211 *
4.	Comfort mandibular denture	4.64 ± 1.245	5.49 ±1.198	7.96 ± 0.918	8.36 ± 1.143	0.0000 *	0.1275
5.	Speech	5 ± 1.118	5.4 ± 0.882	8.21 ± 0.780	8.24 ± 1.114	0.0000 *	0.1244
6.	Ease of chewing	4.72 ± 1.125	5.3 ± 1.113	7.36 ± 0.895	7.85 ± 1.078	0.0000 *	0.0388 *
7.	Esthetics	5.15 ± 1.121	4.64 ± 1.087	8.51 ± 0.667	8.34 ± 1.121	0.0000 *	0.0126 *
8.	General Satisfaction	5.03 ± 1.103	4.09 ± 1.112	7.90 ± 0.630	8.46 ± 0.786	0.0000 *	0.0473 *

Abbreviation: GP = Group, SD = standard deviation, M = number of months; S: significance level (*p* < 0.05); *: indicates significant differences. In the unpaired “*t*” test the level of the degree of significance was determined on the value of *p* ˂ 0. 05. Degree of linear relationship between two variables determined by the Karl Pearson correlation coefficient expressed as r.

**Table 4:** Outline of home care instructions (for elderly patient and FCG) to sustain the influence of psychotherapeutic education for the long term.

1. FCG must assist/enquire/plan for the elderly patient’s daily activities (minimum of once/day).
2. The FCG must plan and organize the caregiving responsible distribution among his/her family members.
3. The FCG must encourage the patient to have a healthy lifestyle that includes self-care, adhering to treatment, and adhering to and following post-insertion instructions.
4. The FCG must remind/give/provide medications for the elderly if and when desired.
5. Any medical/dental equipment/ tool (such as diabetic kit, blood pressure unit) should be operated by the FCG him- or herself. 6. The FCG must instruct on/prepare/bring the constituents for a special diet for the elderly.
7. The FCG must facilitate family understanding and remove conflicts within/outside the family, or family members including relatives, friends and neighbors.
8. The FCG must at all times be the one who will communicate with the concerned doctor as part of the long-term maintenance.
9. The FCG must sit with the elderly patient and facilitate the patient’s understanding regarding complex post-treatment instructions.
10. The FCG must arrange or make all necessary appointments with the concerned healthcare workers.
11. The FCG must try to negotiate with other caregivers within the family and describe their roles on a daily/weekly/ monthly/ annual basis.
12. The elderly patient must at all times appreciate the concerns raised by the FCG.
13. The elderly patient must surrender his/her will and not dictate things that are related to the treatment care.
14. The elderly patient must disclose his/her difficulties to the FCG either directly or indirectly.
15. The elderly patient must allow the FCG to take/make decisions on his/her behalf.
16. The elderly patient must at all times takes positive steps to initiate self-care rather than wait for someone to provide the care.

## Data Availability

Mentioned in supplementary materials.

## Supplementary Materials

The following are available online at https://www.mdpi.com/article/10.3390/healthcare9050533/s1.

## References

[1] Soneja S. (2001). Elder Abuse in India: Country Report for World Health Organization.

[2] Randel J., German T., Ewing D. (1999). The Ageing and Development Report: Poverty, Independence and the World’s Older People.

[3] Council on Scientific Affairs (1987). Elder abuse and neglect. JAMA.

[4] Atetwe L.K., Shankardass M. (2020). Prevalence of elder abuse in Emuhaya sub-county, Vihiga County, Kenya. International Handbook of Elder Abuse and Mistreatment.

[5] Lowenstein A., Eisikovits Z., Band-Winterstein T., Enosh G. (2009). Is elder abuse and neglect a social phenomenon? Data from the First National Prevalence Survey in Israel. J. Elder Abuse Negl..

[6] Yon Y., Mikton C.R., Gassoumis Z.D., Wilber K.H. (2017). Elder abuse prevalence in community settings: A systematic review and meta-analysis. Lancet Global Health.

[7] Sooryanarayana R., Choo W.Y., Hairi N.N. (2013). A review on the prevalence and measurement of elder abuse in the community. Trauma Violence Abus..

[8] Mattoo K.A., Shalabh K., Khan A. (2010). Geriatric forensics: A dentist′s perspective and contribution to identify existence of elder abuse among his patients. J. Forensic Dent. Sci..

[9] Mattoo K.A., Garg R., Dhingra S. (2019). Classifying elder abuse—A review. Gerontol. Geriatr. Res..

[10] Shubayr M.A., Mattoo K.A. (2020). Parental neglect of feeding in obese individuals. A review of scientific evidence and its application among Saudi population. Saudi Med. J..

[11] Okoye U.O., Asa S.S. (2011). Caregiving and stress: Experience of people taking care of elderly relations in south-eastern Nigeria. Arts Soc. Sci. J..

[12] Epstein R.M. (2003). Virtual physicians, health systems, and the healing relationship. J. Gen. Intern. Med..

[13] Fulmer T., Paveza G., Abraham I., Fairchild S. (2000). Elder neglect assessment in the emergency department. J. Emerg. Nurs..

[14] Garg R., Mattoo K., Kumar L., Khalid I., Baig F., Elnager M., Faridi M.A. (2021). Impact of Sensitization of Family Caregivers upon Treatment Compliance among Geriatric Patients Suffering from Elder Abuse and Neglect. Healthcare.

[15] Dong X., Simon M.A. (2013). Elder abuse as a risk factor for hospitalization in older persons. JAMA Intern Med..

[16] Schofield M.J., Powers J.R., Loxton D. (2013). Mortality and disability outcomes of self-reported elder abuse: A 12-year prospective investigation. J. Am. Geriatr. Soc..

[17] Mattoo K.A., Garg R., Kumar S. (2015). Geriatric forensics—Part 2. Prevalence of elder abuse and their potential forensic markers among medical and dental patients. J. Forensic Dent. Sci..

[18] Dong X., Simon M.A., Odwazny R., Gorbien M. (2008). Depression and elder abuse and neglect among a community-dwelling Chinese elderly population. J. Elder Abuse Negl..

[19] DiMatteo M.R., Lepper H.S., Croghan T.W. (2000). Depression is a risk factor for noncompliance with medical treatment: Meta-analysis of the effects of anxiety and depression on patient adherence. Arch. Intern. Med..

[20] John M.T., Micheelis W., Steele J.G. (2007). Depression as a risk factor for denture dissatisfaction. J. Dent. Res..

[21] Johnson T.F., Pillemer K., Wolf R. (1986). Critical issues in the definition of Elder mistreatment. Elder Abuse: Conflict in the Family.

[22] Kua E.H., Ko S.M. (1992). A questionnaire to screen for cognitive impairment among elderly people in developing countries. Acta Psychiatr. Scand..

[23] Yaffe M.J., Tazkarji B. (2012). Understanding elder abuse in family practice. Can. Fam. Physician.

[24] Jeganathan S., Thean H.P., Thong K.T., Chan Y.C., Singh M. (1996). A clinically viable index for quantifying denture plaque. Quintessence Int..

[25] McCunniff M., Liu W., Dawson D., Marchini L. (2017). Patients’ esthetic expectations and satisfaction with complete dentures. J. Prosthet. Dent..

[26] Wolf R.S., Daichman L., Bennett G., Krug E.E., Dahlberg L.L., Mercy J.A., Zwi A.B., Lozano R. (2002). Abuse of the elderly. World Report on Violence and Health.

[27] Mattoo K.A., Shalabh K., Khan A. (2009). Prevelance of elder abuse among completely edentulous patients seeking complete denture prosthesis—A survey. J. Indian Acad. Geriatr..

[28] Hoover R.M., Polson M. (2014). Detecting elder abuse and neglect: Assessment and intervention. Am. Fam. Physician.

[29] Amstadter A.B., Begle A.M., Cisler J.M., Hernandez M.A., Muzzy W., Acierno R. (2010). Prevalence and correlates of poor self-rated health in the United States: The national elder mistreatment study. Am. J. Geriatr. Psychiatry.

[30] Platts-Mills T.F., Barrio K., Isenberg E.E., Glickman L.T. (2014). Emergency physician identification of a cluster of elder abuse in nursing home residents. Ann. Emerg. Med..

[31] Ahmed A., Choo W.Y., Othman S., Hairi N.N., Hairi F.M., Mydin F.H.M., Jaafar S.N.I. (2016). Understanding of elder abuse and neglect among healthcare professionals in Malaysia: An exploratory survey. J. Elder Abus. Negl..

[32] McLellan A. (2008). Nurses have a key role to play in tackling elder abuse. Nurs. Times.

[33] Dyer C.B., Pavlik V.N., Murphy K.P., Hyman D.J. (2000). The high prevalence of depression and dementia in elder abuse or neglect. J. Am. Geriatr. Soc..

[34] Wiseman M. (2008). The role of the dentist in recognizing elder abuse. J. Can. Dent. Assoc..

[35] Jablonski R.A., Munro C.L., Grap M.J., Schubert C.M., Ligon M., Spigelmyer P. (2009). Mouth Care in Nursing Homes: Knowledge, Beliefs, and Practices of Nursing Assistants. Geriatr. Nurs..

[36] Zuluaga D.J., Ferreira J., Montoya J.A., Willumsen T. (2012). Oral health in institutionalised elderly people in Oslo, Norway and its relationship with dependence and cognitive impairment. Gerodontology.

[37] Day A., Boni N., Evert H., Knight T. (2017). An assessment of interventions that target risk factors for elder abuse. Health Soc. Care Community.

[38] Rosen T., Makaroun L.K., Conwell Y., Betz M. (2019). Violence in older adults: Scope, im-pact, challenges, and strategies for prevention. Health Aff..

[39] Van Den Bruele A.B., Dimachk M., Crandall M. (2019). Elder abuse. Clin. Geriatr. Med..

[40] Marshall K., Herbst J., Girod C., Annor F. (2020). Do interventions to prevent or stop abuse and neglect among older adults work? A systematic review of reviews. J. Elder Abuse Neglect..

[41] Covinsky K.E., Fortinsky R.H., Palmer R.M., Kresevic D.M., Landefeld C.S. (1997). Relation between symptoms of depression and health status outcomes in acutely ill hospitalized older persons. Ann. Intern. Med..

[42] Wells K.B., Rogers W., Burnam A., Greenfield S., Ware J.E. (1991). How the medical comorbidity of depressed patients differs across health care settings: Results from the Medical Outcomes Study. Am. J. Psychiatry.

[43] Coulehan J.L., Schulberg H.C., Block M.R., Janosky J.E., Arena V.C. (1990). Medical comorbidity of major depressive disorder in a primary medical practice. Arch. Intern Med..

[44] Roberto K.A. (2016). The complexities of elder abuse. Am. Psychol..

[45] Storey J.E. (2020). Risk factors for elder abuse and neglect: A review of the literature. Aggress. Violent Behav..

[46] Douglas K.S., Skeem J.L. (2005). Violence risk assessment: Getting specific about being dynamic. Psychol. Public Policy Law.

[47] Gitlin L.N., Wolff J. (2012). Family involvement in care transitions of older adults: What do we know and where do we go from here?. Annu. Rev. Gerontol. Geriatr..

[48] Wolff J.L., Spillman B.C., Freedman V.A., Kasper J.D. (2016). A national profile of family and unpaid caregivers who assist older adults with health care activities. JAMA Intern. Med..

[49] King R.B., Ainsworth C.R., Ronen M., Hartke R.J. (2010). Stroke caregivers: Pressing problems reported during the first months of caregiving. J. Neurosci. Nurs..

[50] Roth D.L., Fredman L., Haley W.E. (2015). Informal caregiving and its impact on health: A reappraisal from population-based studies. Gerontologist.

[51] Kim J.H., Knight B.G., Longmire C.V. (2007). The role of families in stress and coping processes among African American and white dementia caregivers: Effects on mental and physical health. Health Psychol..

